# Fabrication of a Plasmonic Nanoantenna Array Using Metal Deposition on Polymer Nanoimprinted Nanodots for an Enhanced Fluorescence Substrate

**DOI:** 10.3390/polym13010048

**Published:** 2020-12-25

**Authors:** Jun Kim, Naseem Abbas, Seongmin Lee, Jeongwoo Yeom, Md Ali Asgar, Mohsin Ali Badshah, Xun Lu, Young Kyu Kim, Seok-Min Kim

**Affiliations:** 1 Department of Mechanical Engineering, Chung-Ang University, Seoul 06974, Korea; zuhn@cau.ac.kr (J.K.); naseem@cau.ac.kr (N.A.); papillon14@cau.ac.kr (J.Y.); mohsinali@cau.ac.kr (M.A.B.); luxun@ybu.edu.cn (X.L.); 2 Department of Mechanical System Engineering, Chung-Ang University, Seoul 06974, Korea; min0312@cau.ac.kr; 3 Department of Computer Science and Engineering, Chung-Ang University, Seoul 06974, Korea; asgar@cau.ac.kr (M.A.A.); kykdes@cau.ac.kr (Y.K.K.); 4 Department of Mechanical Engineering, Yanbian University, Yanji 133002, China

**Keywords:** polymer nanoimprinting, plasmonic nanoantenna array, metal-enhanced fluorescence, localized surface plasmon resonance

## Abstract

A simple and cost-effective method is proposed herein for a plasmonic nanoantenna array (PNAA) for the fabrication of metal-enhanced fluorescence (MEF) substrates in which fluorophores interact with the enhanced electromagnetic field generated by a localized surface plasmon to provide a higher fluorescence signal. The PNAA is fabricated by the deposition of a silver (Ag) layer on an ultraviolet (UV) nanoimprinted nanodot array with a pitch of 400 nm, diameter of 200 nm, and height of 100 nm. During deposition, raised Ag nanodisks and a lower Ag layer are, respectively, formed on the top and bottom of the imprinted nanodot array, and the gap between these Ag layers acts as a plasmonic nanoantenna. Since the thickness of the gap within the PNAA is influenced by the thickness of Ag deposition, the effects of the latter upon the geometrical properties of the fabricated PNAA are examined, and the electromagnetic field intensity distributions of PNAAs with various Ag thicknesses are simulated. Finally, the fluorescence enhancement factor (FEF) of the fabricated PNAA MEF substrate is measured using spotted Cy5-conjugated streptavidin to indicate a maximum enhancement factor of ~22× for the PNAA with an Ag layer thickness of 75 nm. The experimental results are shown to match the simulated results.

## 1. Introduction

Fluorescence-based detection technology has been widely used for the analysis of protein and DNA microarray biosensors. The application of metal-enhanced fluorescence (MEF), in which fluorophores interact with metallic nanostructures to enhance fluorescence, is regarded as a powerful approach to improving the sensitivity of microarray biosensors [1,2,3,4]. Metallic nanostructures can enhance fluorescence because the localized surface plasmon effect enhances their surrounding electromagnetic (EM) fields [5,6,7].

To apply the MEF technique to microarray biosensors, a low-cost method for the fabrication of a uniform metallic nanostructure on a 25 × 75 mm^2^ glass slide substrate with a high fluorescence enhancement factor (FEF) is required. To realize a low-cost, large-area MEF substrate, methods for the fabrication of random nanostructures have been studied, including electrochemistry [8,9], wet chemistry [10,11], chemical deposition [12,13], physical vapor deposition [1,2,3,14], and solution-seeded nanorod synthesis [15]. However, these processes are limited in terms of shape controllability and uniformity. A theoretically designed metallic nanostructure is required for maximizing the FEF due to its high dependence upon the geometrical properties of the metallic nanostructure (size, shape, density, height, gap, etc.) [16,17,18,19,20]. In addition, a uniform FEF throughout the MEF substrate is important for microarray applications. In this respect, nanostructuring techniques such as electron beam lithography [21,22,23] and focused ion beam machining [24] are suitable for fabricating nanostructures with high uniformity on relatively large areas. For maximal FEF, MEF nanostructures with closely located discrete metallic nanostructures, termed nanoantenna structures, have been reported. For instance, Bakker et al. used electron beam lithography to fabricate Au elliptical geometries, which worked as plasmonic nanoantenna MEF substrates and exhibited FEFs of 10 to 70, depending on the nanoantenna gap [21,22]. The same technique was used by Kinkhabwala et al. to produce Au bowtie nanoantennas that offer single-molecule detection with a maximum FEF of 1340 [23]. Similarly, Punj et al. fabricated Au antenna-in-box platforms with FEFs of up to 1100 [24]. Although these techniques have provided FEFs via precisely controlled nanostructures, they are too expensive for application to extending the size of the nanostructured MEF substrates to that of a 25 × 75 mm^2^ glass slide. Although microsphere lithography [25,26] and laser interference lithography [27] have been investigated for the fabrication of large-area MEF substrates, these processes remain limited in terms of shape controllability and large-area uniformity.

To date, nanoimprint lithography is considered to be the most suitable, low-cost, and large-area nanofabrication technique for MEF substrates. In this technique, a polymeric nonpattern is replicated from the nanostructured master pattern and used as an etch barrier or final nanostructure. For example, Yoo et al. used reactive ion etching with a thermal nanoimprinted nanodot etch barrier pattern to fabricate a highly ordered Ag nanodot array MEF substrate with an FEF of 15 for the detection of Cy3 fluorophores [28]. Similarly, Zhang et al. fabricated a plasmonic nanodot antenna by depositing an Au layer on a reactive ion-etched SiO_2_ nanodot array with a nanoimprinted barrier pattern and achieved an FEF of 2970 [29]. Although the nanoimprinted polymer pattern can be used as an etch barrier of metallic or glass material, the polymeric nanostructure is also durable to apply to one-time-use biosensors. Therefore, the imprinted polymeric nanostructure can be used as a scaffold for the metallic nanostructure. Akashi et al. [30] and Badshah et al. [31] produced MEF substrates by the deposition of Ag layers on nanoimprinted polymer nanostructure patterns in a simple and cost-effective approach that does not require an additional etching process. Nevertheless, this process cannot provide a nanoantenna structure because a continuous metallic layer is deposited on the imprinted nanostructure.

In the present study, a three-dimensional plasmonic nanoantenna array (PNAA) was realized as an MEF substrate by the deposition of an Ag layer on a nanoimprinted polymer nanodot array. During deposition, Ag nanodisks and an Ag layer were, respectively, formed on the top and bottom of the imprinted nanodot array, and the gap between these layers acts as a plasmonic nanoantenna. A KrF laser scanning lithographed eight-inch silicon master, equipped with a nanodot array with a height of 100 nm, diameter of 200 nm, and pitch of 400 nm, was used. A polymer nanodot array with dimensions similar to those of the Si master was fabricated by nanoimprinting on a 25 × 75 mm^2^ glass slide substrate with a replicated polymer nanohole template. To realize the PNAA, an Ag layer was deposited on the imprinted polymer nanodot array. To optimize the thickness of the Ag layer and maximize the FEF, the effects of the deposited Ag layer thickness upon the simulated EM fields and the measured fluorescence signals of a spotted Cy5-conjugated streptavidin (SA-Cy5) on the PNAA substrate were analyzed.

## 2. Fabrication of Plasmonic Nanoantenna Array (PNAA) Substrate

**Figure 1:**
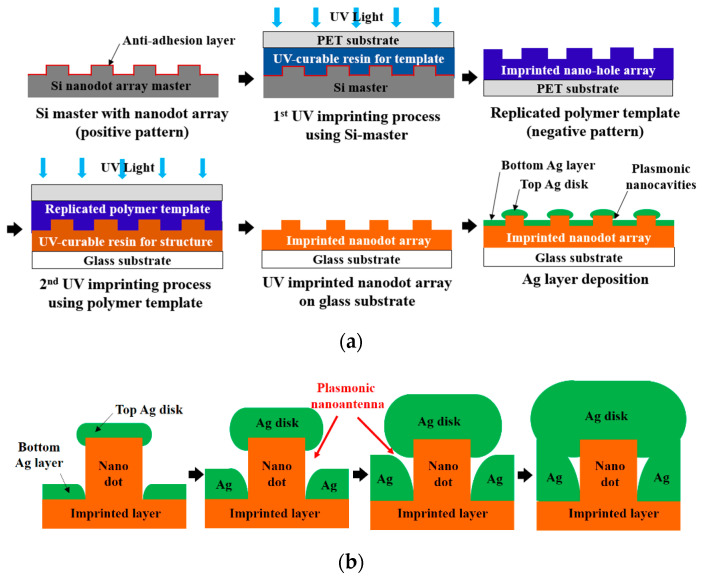
Schematic diagrams of (**a**) the fabrication process for the proposed plasmonic nanoantenna array (PNAA) via UV nanoimprinting and Ag deposition, and (**b**) the plasmonic nanoantenna formation process with increasing Ag deposition thickness.

A PNAA MEF substrate was fabricated by a UV nanoimprinting and Ag deposition process on a 25 × 75 mm^2^ glass slide substrate, as shown schematically in Figure 1a. An eight-inch silicon master containing the nanodot array was fabricated via KrF laser scanning photolithography (NSR-S203B, Nikon Co., Ltd., Tokyo, Japan) and reactive ion etching by following a previously reported procedure [32]. The designed diameter, pitch, and height of the nanodot array silicon master were 200, 400, and 100 nm, respectively. To prevent adhesion during UV nanoimprinting, a self-assembled monolayer was first applied by dipping the silicon master in a 2% solution of dimethyldichlorosilane in octamethylcyclotetrasiloxane (Repel-Silane ES, GE Healthcare Co., Ltd., Chicago, IL, USA). To fabricate the polymer nanodot array on the glass slide substrate, a transparent polymer template was replicated from the Si master via the UV-nanoimprinting process. In this process, a UV-curable urethane acrylate-based photopolymer (KR001, EONANOCHEM Co., Ltd., Sejong, Korea), which was formulated to provide the template material with a good releasing property, was dispensed onto the Si nanodot array master in several small droplets, and then covered with a primer-treated polyethylene terephthalate (PET) film (SH34, SKC Co., Ltd., Seoul, Korea). The UV-curable resin was then squeezed into a uniform thin film between the silicon and PET film via the roll pressing method. The PET film was then irradiated with high-intensity UV light to cure the photopolymer material, after which the transparent polymer template with the replicated nanohole array was separated from the Si master. The substrate was then cleaned with isopropyl alcohol and de-ionized water and coated with trialkoxysilane solution (Exfix ZAP-1020, Chem-Optics Co., Ltd., Daejeon, Korea) to increase the adhesion properties, followed by a second imprinting process using a different UV-curable urethane acrylate-based photopolymer (AA3040, SK Chemicals Co., Ltd., Seongnam, Korea) to provide high mechanical strength and optical transparency. In this step, the UV-curable resin was dropped onto a glass slide substrate and covered by the previously replicated polymer template. After the UV irradiation, the UV-imprinted nanodot array on a glass slide substrate was obtained.

**Figure 2:**
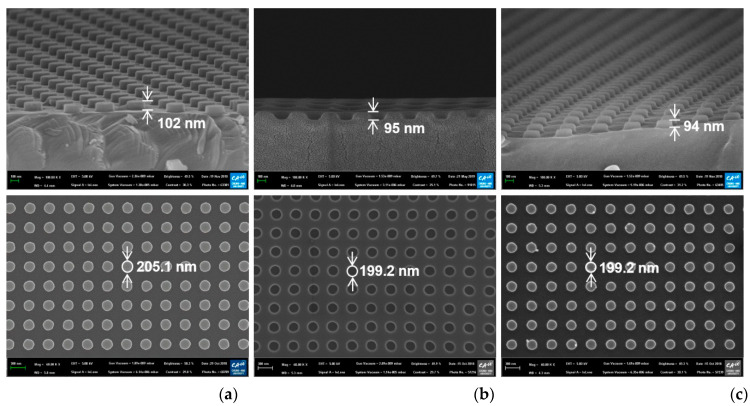
Top-view (bottom) and cross-sectional (top) scanning electron microscope (SEM) images of (**a**) the silicon master, (**b**) the polymer template, and (**c**) the UV-imprinted nanodot pattern.

Top-view and cross-sectional scanning electron microscope (SEM) images of the fabricated silicon nanodot master, the UV-imprinted polymer nanohole template, and the UV-imprinted nanodot array on the glass slide substrate are presented in Figure 2. While all the samples had a pitch of 400 nm, the diameters of the silicon master, polymer template, and nanoimprinted pattern were 205.1, 199.2, and 199.2 nm, respectively (bottom row, Figure 2). In addition, the corresponding heights are seen to be 102, 95, and 94 nm, respectively (top row, Figure 2). The slight differences between the designed diameter (200 nm) and height (100 nm) and the measured values in the master pattern (205.1 and 102 nm) might be due to measurement errors and fabrication errors in the reactive ion etching process. The difference in diameter and height between the master pattern and imprinted nanodots might be due to the shrinkage of UV-imprinted resin during the curing process.

Although Au and Ag are both commonly used as materials for MEF substrates, Ag was selected for the PNAA MEF substrate in the present study due to its cost-effectiveness. Although the natural oxidation of Ag might deteriorate the fluorescence enhancement, the thickness of the natural oxide layer is just a few nanometers, and it does not significantly degrade the enhanced EM field [33]. The Ag layer was deposited onto the polymer imprinted nanodot array on the glass substrate via electron-beam (E-beam) evaporation at a rate of 0.3 Å/s under a vacuum condition of ~9.5 × 10^−7^ Torr. The fabrication of the plasmonic nanoantenna via deposition of an Ag layer onto the imprinted nanodot array is shown schematically in Figure 1b. During deposition, an Ag nanodisk formed on top of the imprinted nanodots, and a lower Ag layer deposited in the spaces between the nanodots in the array. As the gap between the Ag nanodisks and the lower Ag layer decreases with increasing Ag deposition thickness, a sufficiently narrow gap could be obtained to provide the nanoantenna for the MEF substrate. With a further increase in the Ag deposition thickness, the Ag nanodisk and the lower Ag layer connected and the plasmonic nanoantenna disappeared. Hence, the effects of the Ag deposition thickness upon the geometrical properties of the fabricated PNAA were examined.

**Figure 3:**
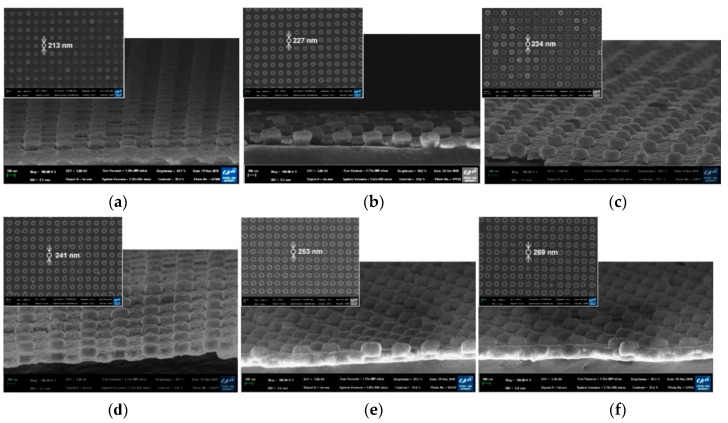
Cross-sectional SEM images of the fabricated PNAA MEF substrates with Ag thicknesses of (**a**) 25 nm, (**b**) 50 nm, (**c**) 75 nm, (**d**) 100, (**e**) 125, and (**f**) 150 nm. The insets show the corresponding top-view SEM images. Enlarged cross-sectional SEM images of Figure 3a–d are included in the Supplementary Materials.

The cross-sectional SEM images of the fabricated PNAA with the target Ag layer thicknesses of 25, 50, 75, 100, 125, and 150 nm are presented in Figure 3, along with the corresponding top-view SEM images of the PNAA (top left insets). By increasing the Ag deposition thickness, the diameters of the Ag disks on top of the imprinted nanodots increased from 213 to 269 nm due to side-wall deposition during the E-beam evaporation process. In addition, the Ag disks appear to be thicker than the deposition thickness of the Ag layer because the upper section of the nanodot side wall was also coated with Ag, as shown schematically in Figure 1b. Plasmonic nanogaps were generated between top Ag disks and bottom Ag layers, and the gap decreased as the Ag deposition thickness increased, as shown in the enlarged cross-sectional SEM images of Figure 3 in the Supplementary Materials (Figures S1–S4). The side-wall deposition effect is shown in Figure 3b and Figure S2, where the sample is cut to reveal the cross-sectional structure. Here, the total thickness of the nanodot and Ag disk is ~150 nm, thus suggesting that an ~50 nm Ag layer was deposited onto the 100 nm nanodots, as expected. However, the thickness of the top Ag disk was found to be ~70 nm, and the height of the underlying nanodot was ~80 nm. With a further increase in the Ag deposition thickness, the plasmonic gap decreased (Figure 3c and Figure S3) and disappeared when the deposition thickness was higher than 100 nm (Figure 3d and Figure S4). Thus, the narrowest gap is visible in Figure 3c, where the deposition thickness is 75 nm.

## 3. Analysis of Enhanced Electromagnetic Field Distribution of the PNAA Substrate

**Figure 4:**
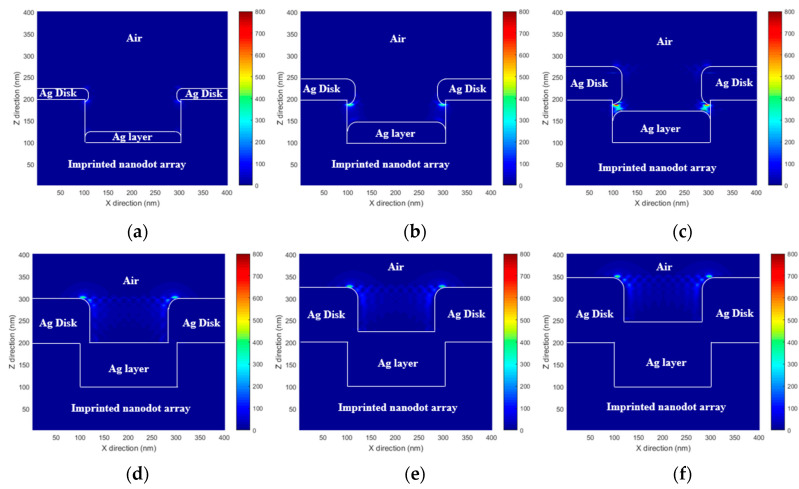
Simulated *x*–*z* plane (*y* = 200 nm) electromagnetic field intensity distributions at the PNAA base plane of PNAA MEF substrates with Ag deposition thicknesses of (**a**) 25, (**b**) 50, (**c**) 75, (**d**) 100, (**e**) 125, and (**f**) 150 nm. The color bar represents the electromagnetic field intensity (${\left|E\right|}^{2}$).

To predict the performance of the proposed MEF substrate, the enhanced EM field distribution of the PNAA was calculated via rigorous coupled wave analysis (RCWA). A commercial RCWA software package (RSoft-DiffractMOD, Synopsys, Inc., Mountain View, CA, USA) was used for the simulation. A simplified 3-dimensional PNAA structure model was constructed. It consisted of imprinted nanodots (*n* = 1.482) with a diameter of 200 nm and a height of 100 nm, a top Ag disk (*n* = 0.056 + 4.29i) with the measured diameter and height, and a lower Ag layer (*n* = 0.056 + 4.29i) with the measured height. In the simplified simulation model, the sides of both the top Ag disk and the lower Ag layer were considered to be rounded, as shown in Figure 4. In the RCWA simulation, a *y*-direction polarized light with a wavelength of 635 nm illuminated the PNAA from the vertical direction, and one unit volume (400 × 400 × 400 nm) was set as the simulation region under the periodic *x*- and *y*-direction boundary conditions. A background refractive index of 1 was set for air. Finally, an EM intensity distribution was simulated to explain the hotspot regions in the PNAA. The EM intensity was calculated according to the square power of the EM amplitude.

The RCWA-simulated *x*–*z* plane EM field intensity distributions at *y* = 200 nm for PNAA MEF substrates with Ag deposition thicknesses of 25, 50, 75, 100, 125, and 150 nm are presented in Figure 4. In each case, the maximum EM field intensity is generated around the top Ag disk. In Figure 4a, high EM fields are generated at both the top and the bottom edges of the Ag disk because the gap between the top Ag disk and the lower Ag layer is too large to generate a nanoantenna. In Figure 4b,c, the maximum EM field is generated at the gap, which functions as the plasmonic nanoantenna. The highest EM fields are observed in Figure 4c because the smallest gap is obtained. In Figure 4d–f, high EM fields are generated at the top edge of the Ag disk because the bottom edge has disappeared due to connection with the lower Ag layer. This result clearly shows that the maximum EM field intensity is achieved in the PNAA MEF substrate with 75 nm thick Ag layers, which provide the minimum gap width.

## 4. Analysis of the Fluorescence Enhancement Factor (FEF) of the PNAA

To examine the FEF of the PNAA MEF substrates, a nanodispenser (PipeJet^®^, BioFluidixGmbH, Ltd., Freiburg, Germany) was used to spot streptavidin-Cy5 fluorophores (SA-Cy5, Thermo-Fisher Scientific, Waltham, MA, USA) onto the various fabricated PNAA substrates on a motorized *x*–*y* stage. Their fluorescence signals were then measured using a microarray scanner (GenePix 4000B, Molecular Device LLC, San Jose, CA, USA) with an excitation laser wavelength of 635 nm and compared with the signals obtained from the bare glass substrate. Prior to use, the SA-Cy5 was diluted in phosphate buffer silane (PBS, GE Healthcare Co., Ltd., Chicago, IL, USA) to a concentration of 0.01 mg/mL, and 14 spots with a volume of 12 nL each were then applied to the substrate.

**Figure 5:**
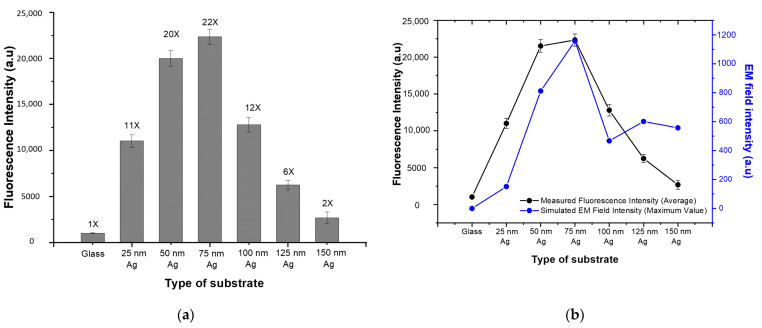
Comparisons of (**a**) the measured fluorescence intensity of the bare glass substrate and the various PNAA MEF substrates (the number above each vertical bar is the calculated fluorescence enhancement factor), and (**b**) the average measured fluorescence intensity and the maximum simulated EM field intensity.

For quantitative analysis, the mean fluorescence intensity was calculated for each spot and defined as the fluorescence signal. The average fluorescence signal of the bare glass substrate and the various PNAA MEF substrates are compared in Figure 5a, where the vertical bars represent the average values and the error bars denote the standard deviations for the 14 spots on the substrate. Since the same amount of SA-Cy5 was spotted on each substrate, the FEF was calculated by dividing the fluorescence signal of the PNAA MEF substrate into that of the glass substrate. The resulting FEF values for each substrate are indicated above the vertical bars. These results indicate that the FEF increases to a maximum of ~22 as the thickness of Ag deposition increases to 75 nm, and then decreases at greater thicknesses. The maximum FEF was obtained when the plasmonic gaps between the top Ag disks and the lower Ag layers are at the optimum width. The measured average fluorescence signals are compared with the simulated maximum EM field intensities for each substrate in Figure 5b. Here, although the basic trends are well matched, the measured trend was found to be higher than the simulated one at Ag deposition thicknesses of 25, 50 and 100 nm. This is because, in contrast to the simplified ideal models used in the simulation, the actual fabricated Ag nanodisks and Ag layers shown in Figure 3 exhibited irregularities and randomness that can generate higher EM field intensities than those indicated by the smooth simulation model and structure. In contrast, the simulated trend was higher than the measured one at Ag deposition thicknesses of 125 and 150 nm because the surface profile of the deposited layer smoothed as the deposition thickness increased, thus decreasing the EF field intensity in the actual sample. In the RCWA simulation of the PNAA with Ag thicknesses greater than 100 nm, the maximum EM field intensity can increase due to the decrease in the distance between neighboring Ag nanodisks and can decrease due to the total thickness of the Ag layer. Therefore, the simulated maximum EM field intensity is not much changed at a deposition thickness greater than 100 nm.

## 5. Conclusions

A UV-imprinted polymeric nanodot array was demonstrated as a scaffold of a metallic nanostructure due to its cost-effectiveness in the fabrication of large-area nanostructures. The MEF substrate consisting of three-dimensional PNAAs with narrow nanogaps was achieved via UV-nanoimprinting and Ag vapor deposition processes. A UV-imprinted polymer nanodot array with a diameter of 200 nm, height of 100 nm, and pitch of 400 nm was fabricated on a glass slide substrate. During deposition, Ag nanodisks formed on the tops of the nanodots, and a lower Ag layer formed between the nanodots in the imprinted array. This resulted in a confined, enhanced EM field at the plasmonic nanogaps obtained at Ag deposition thicknesses of 50 and 75 nm. The maximum FEF of ~22 was experimentally measured at the Ag deposition thickness of 75 nm, where the narrowest nanogaps were obtained. An RCWA simulation was performed to investigate the EM field distributions of PNAAs with various Ag deposition thicknesses, and an excellent agreement with the experimentally measured FEF results was observed. The narrow width of the nanogaps was found to be a critical factor for the MEF using the PNAA structure. The realization of protein microarray biochips using the proposed PNAA MEF substrate is a subject of ongoing research.

## Supplementary Materials

The following are available online at https://www.mdpi.com/2073-4360/13/1/48/s1, Figure S1. Enlarged cross-sectional SEM images of the fabricated plasmonic nanoantenna dot array MEF substrate with the target Ag thicknesses of 25 nm; Figure S2. Enlarged cross-sectional SEM images of the fabricated plasmonic nanoantenna dot array MEF substrate with the target Ag thicknesses of 50 nm; Figure S3. Enlarged cross-sectional SEM images of the fabricated plasmonic nanoantenna dot array MEF substrate with the target Ag thicknesses of 75 nm; Figure S4. Enlarged cross-sectional SEM images of the fabricated plasmonic nanoantenna dot array MEF substrate with the target Ag thicknesses of 100 nm.
