# Supplementary material for: Fabrication of a Plasmonic Nanoantenna Array Using Metal Deposition on Polymer Nanoimprinted Nanodots for an Enhanced Fluorescence Substrate
Source: Polymers (Basel). 2020 Dec 25;13(1):48. doi: 10.3390/polym13010048 (PMC7795982; doi:10.3390/polym13010048)
Supplement: Supplementary file 1 [file polymers-13-00048-s001.pdf]

[Supplementary Materials]

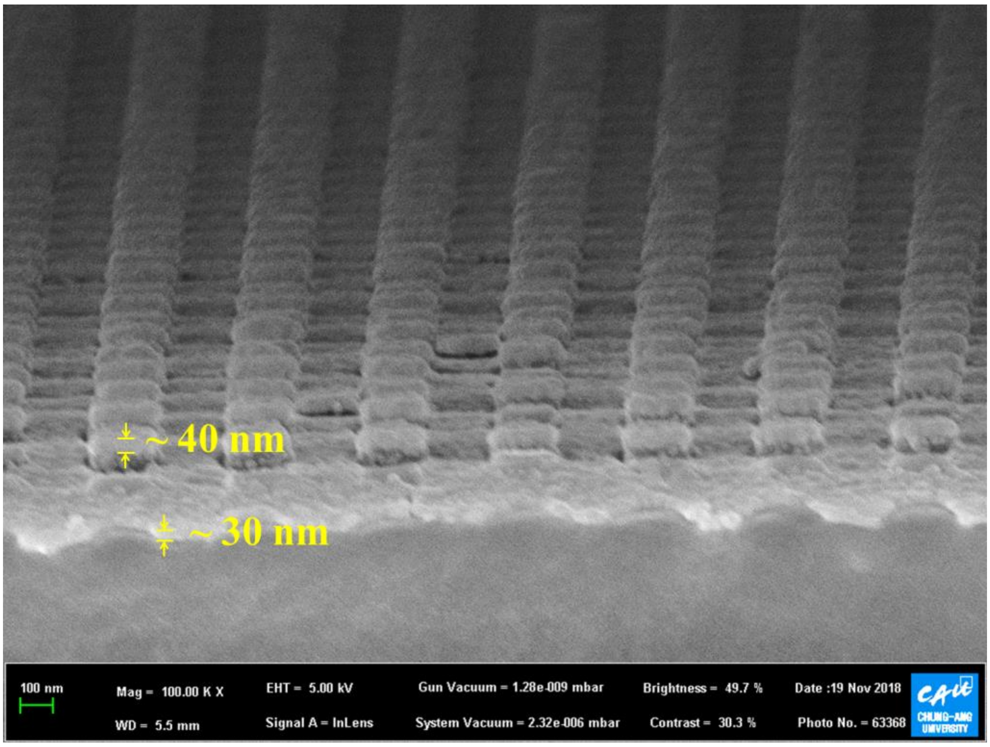

Figure S1. Enlarged cross-sectional SEM images of the fabricated plasmonic nanoantenna dot array MEF substrate with the target Ag thicknesses of 25 nm,

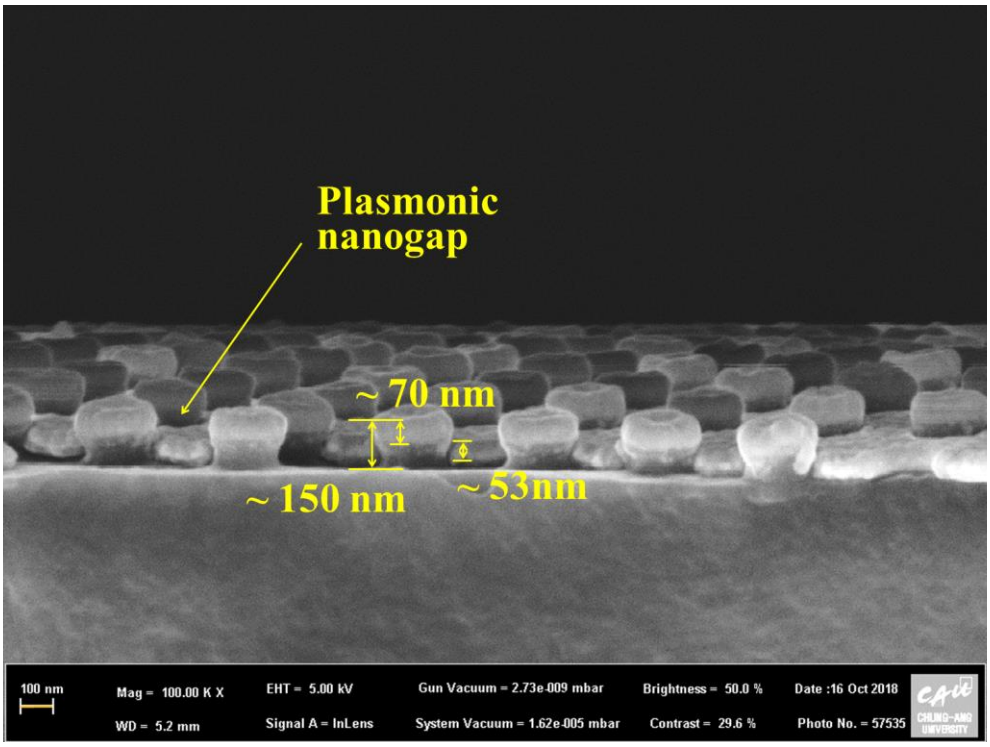

Figure S2. Enlarged cross-sectional SEM images of the fabricated plasmonic nanoantenna dot array MEF substrate with the target Ag thicknesses of 50 nm,

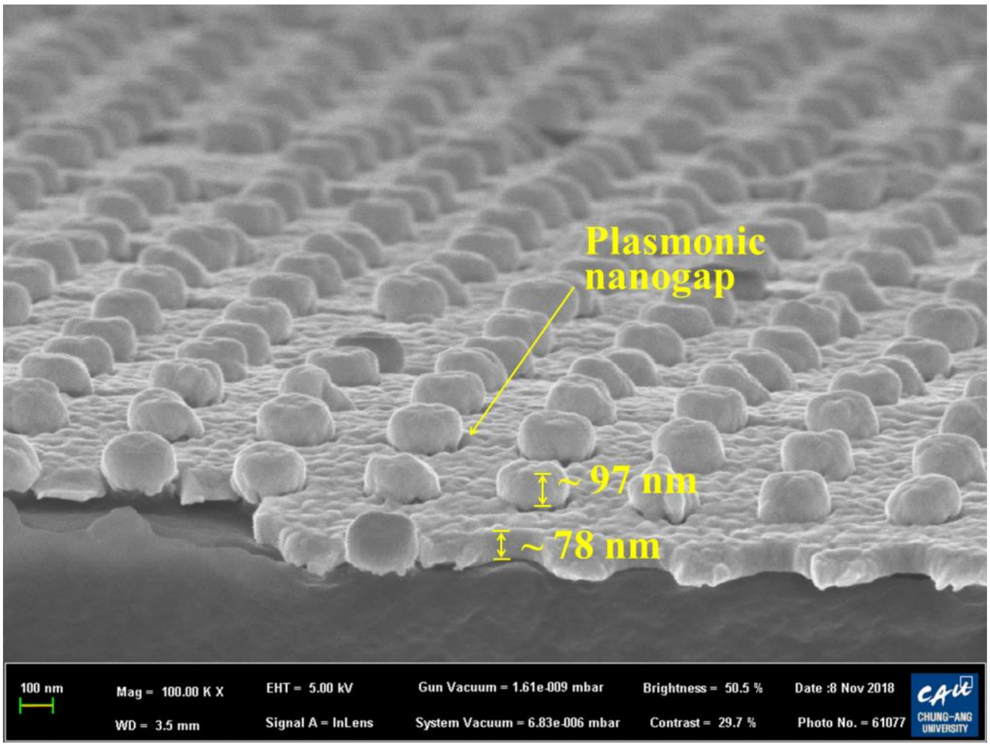

Figure S3. Enlarged cross-sectional SEM images of the fabricated plasmonic nanoantenna dot array MEF substrate with the target Ag thicknesses of 75 nm,

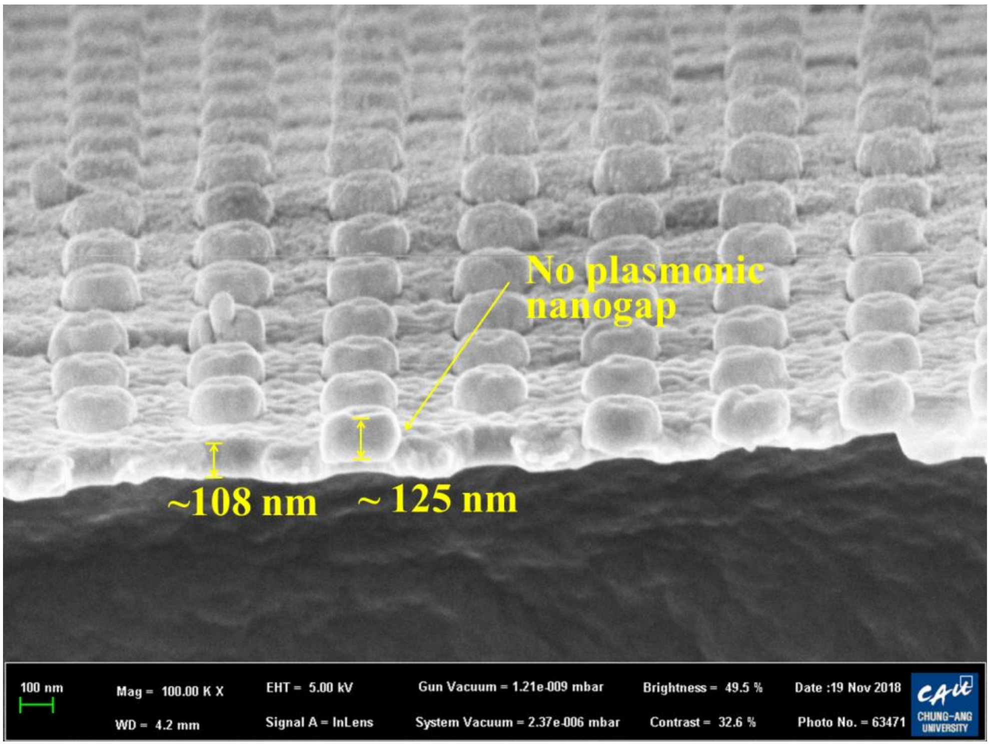

Figure S4. Enlarged cross-sectional SEM images of the fabricated plasmonic nanoantenna dot array MEF substrate with the target Ag thicknesses of 100 nm,
